# The complex lipid, SPPCT-800, reduces lung damage, improves pulmonary function and decreases pro-inflammatory cytokines in the murine LPS-induced acute respiratory distress syndrome (ARDS) model

**DOI:** 10.1080/13880209.2022.2087689

**Published:** 2022-07-03

**Authors:** Peter P. Sordillo, Andrea Allaire, Annie Bouchard, Dan Salvail, Sebastien M. Labbe

**Affiliations:** a SignPath Pharma, Inc, New York, NY, USA; b IPS Therapeutique, Sherbrooke, Quebec, Canada

**Keywords:** Acute respiratory distress syndrome, bronchoalveolar lavage fluid, inflammatory cytokines, lung index, lung injury

## Abstract

**Context:**

Acute respiratory distress syndrome (ARDS) is a highly fatal, inflammatory condition of lungs with multiple causes. There is no adequate treatment.

**Objective:**

Using the murine LPS-induced ARDS model, we investigate SPPCT-800 (a complex lipid) as treatment for ARDS.

**Materials and methods:**

C57B16/N mice received 50 μg of *Escherichia coli* O111:B4 lipopolysaccharide (LPS). SPPCT-800 was given as either: (1) 20 or 200 mg/kg dose 3 h after LPS; (2) 200 mg/kg (prophylactically) 30 min before LPS; or (3) eight 200 mg/kg treatments over 72 h. Controls received saline installations.

**Results:**

At 48 and 72 h, SpO_2_ was 94% and 90% in controls compared to 97% and 94% in treated animals. Expiration times, at 24 and 48 h, were 160 and 137 msec for controls, but 139 and 107 msec with SPPCT-800. In BALF (24 h), cell counts were 4.7 × 10^6^ (controls) and 2.9 × 10^6^ (treated); protein levels were 1.5 mg (controls) and 0.4 mg (treated); and IL-6 was 942 ± 194 pg/mL (controls) versus 850 ± 212 pg/mL (treated) [at 72 h, 4664 ± 2591 pg/mL (controls) versus 276 ± 151 pg/mL (treated)]. Weight losses, at 48 and 72 h, were 20% and 18% (controls), but 14% and 8% (treated). Lung injury scores, at 24 and 72 h, were 1.4 and 3.0 (controls) and 0.3 and 2.2 (treated).

**Discussion and conclusions:**

SPPCT-800 was effective in reducing manifestations of ARDS. SPPCT-800 should be further investigated as therapy for ARDS, especially in longer duration or higher cumulative dose studies.

## Introduction

Acute respiratory distress syndrome (ARDS), characterized by severe hypoxaemia after acute lung injury, is an intense inflammatory process in the lung that results in a high mortality. Etiologic factors include sepsis, pneumonia, acute pancreatitis, chemical or smoke inhalation, aspiration of gastric contents, traumatic shock, chemotherapy toxicity, or viral illnesses including SARS-CoV-2 (Kirch et al. 2003; Dushianthan et al. 2011; Confalonieri et al. 2017; Thompson et al. 2017; Grasselli et al. 2020; Xu et al. 2020). Hypoxaemia secondary to cardiogenic pulmonary edoema is excluded in the diagnosis of ARDS. In patients with ARDS, damage to the lung can begin days after the initial insult, with damage to alveoli and major edoema in the lungs which are manifested by diffuse infiltrates on chest radiograph. After 7–10 days, damage to the lung can progress to fibrosis (Ware and Matthay 2000; Spadaro et al. 2019). Patients with ARDS lung damage have a decreased PaO_2_ (partial pressure of oxygen)/FiO_2_ (fraction of inspired oxygen) ratio, indicating the degree of hypoxaemia. Decreasing PaO_2_/FiO_2_ values indicate worsening lung damage.

Increases in neutrophils are found prominently in the bronchoalveolar lavage fluid (BALF) in these patients, and these cells are important in the progression of this disease (Windsor et al. 1993; Matute-Bello et al. 1997; Juss et al. 2016). Levels of neutrophils, and of neutrophil to lymphocyte ratios, are prognostic indicators in these patients (Wang et al. 2018; Ma et al. 2020), and neutrophil depletion in animal models may partially reduce lung damage (Williams and Chambers 2014). The neutrophils that migrate to the lungs in response to lung inflammation cause the release of pro-inflammatory cytokines. This sets off a cascade of inflammation which increases lung damage (Scott and Kubes 2018; Yang et al. 2020). The neutrophils are known to stimulate particularly the secretion of interleukins (ILs), known to correlate with the severity of ARDS lung damage (Rebetz et al. 2018). The secreted cytokines, in turn, recruit additional neutrophils to the lung (Chen and Kolls 2016). Yang et al. (2020), in a recent review, reported that prominent cytokines, including IL-6, interferon-γ (IFN-γ), and granulocyte colony-stimulating factor (G-CSF) markedly increase in the lung. Others have also reported that these cytokines are particularly important in causing profound inflammation and severe disease (Meduri et al. 1995; Preira et al. 2015; Wilson et al. 2020).

In this study, we have used the murine LPS-induced ARDS model, a well-established and commonly used model for studies of this disease (Bastarache and Blackwell 2009; Aeffner et al. 2015). It ‘duplicates the mechanisms and consequences of ARDS and displays major features of microvascular lung injury, including leukocyte accumulation in lung tissue, pulmonary edoema, profound lung inflammation and mortality’ (Chen et al. 2010). It has been reported that in this model, cytokines such as IL-1β, IL-2, IL-5, IL-6, IL-12, IL-17, vascular endothelial growth factor (VEGF), INF-γ monocyte chemoattract protein-1 (MCP-1, CCL2), keratinocytes-derived chemokine (KC, CXCL1), MIP-1α (macrophage inflammatory protein-1α (CCL3)], and interferon-γ induced protein 10 (IP-10, CXCL-10), were all significantly elevated after 18 h (Juskewitch et al. 2012). Further, it is known that mechanical ventilation, when necessary, will cause additional lung damage and inflammation (Ware and Matthay 2000; Henderson et al. 2017; Spadaro et al. 2019). Thus, suppression of inflammation is key to treating this disease.

SPPCT-800 is a complex lipid we have been researching for many years as a treatment to prevent QT prolongation from QT prolonging medications. Increases in pro-inflammatory cytokines play a major role in the causation of QT prolongation (Sordillo et al. 2015; Sordillo and Sordillo 2021b). To investigate the role of SPPCT-800 as a treatment for ARDS, we used the murine LPS model to measure, in treated and control mice, blood oxygen saturations, lung function parameters, BALF protein and cell counts, pro-inflammatory cytokine levels in the plasma and BALF, body weight losses and lung injury scores determined by a histologist.

## Materials and methods

### Mice

C57Bl6/N mice were obtained from Charles River Laboratories (Montreal, Quebec). Male mice, 20–25 g body weight, were used throughout the studies. Animals were housed in specific pathogen‐free conditions. Animals were euthanized by exsanguination under anaesthesia.

### Ethics approval and consent to participate

Animal studies are reported in compliance with AAALAC (Association for Assessment and Accreditation of Laboratory Animal Care International) guidelines. All experiments were approved by the Institutional Animal Care and Use Committee at IPS Therapeutique, Sherbrooke, Quebec, Canada (IPS SL20200402-1). The study was conducted according to ARRIVE guidelines.

### Animal groupings and housing

All delivered mice were kept for one week as an acclimatization period prior to performing any experiments. Animals were housed at a maximum of two mice per cage under a 12 h light/dark cycle at a temperature of ∼20–22 °C and 40–60% humidity. Food and water were available *ad libitum*. Each cage of mice was blindly assigned for different treatments or maintained as a control group according to the experimental design. The individual who carried out the experiments was not blinded as to treatment, but data analysis and experiments were otherwise blinded to avoid any bias.

### Induction of ARDS

In the ARDS group, mice received *Escherichia coli* O111:B4 lipopolysaccharide [50 μg in 0.05 mL saline, intratracheal (i.t.)]. For intratracheal instillation, mice were slightly anaesthetized with isoflurane.

### Treatment with SPPCT-800

SPPCT-800 was dissolved in water (low dose − 2 mg/mL; high dose − 20 mg/mL). For the 24 h study, two treatment approaches were used. Mice received a single dose of SPPCT-800 (200 mg/kg, per gavage) as prevention (30 min before the LPS instillation), or as a single dose of either 20 or 200 mg/kg as therapy (3 h after the LPS instillation). For the 72 h study, mice received a total of eight treatments of SPPCT-800 (200 mg/kg) starting at 3 h after the LPS instillation. In the control group, the animals received an instillation of saline (0.05 mL, i.t.).

### Oxygen saturation

At 0 h (baseline), 24 h post-LPS instillation, and 48 and 72 h post LPS instillation, arterial blood oxygen saturation (SpO_2_) was recorded on conscious mice. SpO_2_ was read off of a pulse oximeter (STARR Life Sciences MouseOx Plus system, Oakmont, PA) with a mouse collar probe installed at the carotid level. The saturation values were measured in percentages (%).

### Respiratory functions

All mice were introduced to the plethysmograph chamber environment. After the acclimatization period, the functional respiratory parameters were assessed by the whole-body plethysmograph (VivoFlow, SCIREQ, Montreal, Canada) at 0 h (baseline), at 24 h post-LPS instillation, and at 48 and 72 h post-LPS instillation. Each measurement was performed with the mouse placed alone in an unrestrained whole-body plethysmography (WBP) chamber to measure respiratory functions. The WBP trace allowed us to determine specific information regarding the breathing pattern, and to derive important information associated with the development of inflammation. The functional respiratory parameters analysed included: respiratory rate, penH (pulmonary congestion index), and inspiratory/expiratory time measurements. PenH was used as an index of edoema, inflammation and congestion (broncho-restriction) (Lomask 2006).

### Cell counts and protein levels in BALF

The left lung was clamped while 0.9 mL of cold PBS 1X, Protease Inhibitor 1X (SigmaFast®) solution (3/300 µL) was injected so bronchoalveolar lavage fluid (BALF) from the right lobe of the lungs could be collected. The protein assay was performed according to the manufacturer’s instruction BCA (Bicinchoninic acid) Protein Assay (Pierce^TM^ – #23227). Briefly, a dilution factor of 5 was used (1 part of BALF: 4 parts of PBS 1x). Diluted sample (10 µL) was added into the microplate wells. The working reagent (200 µL) was added into each well. The plate was covered, and incubated at 37 °C for 30 min. After a cool-down period, the absorbance at 562 nm was rapidly measured using a monochromatic spectrophotometer (SpectraMAX^®^plus – Molecular Devices). The total BALF protein content was reported by multiplying the protein concentration by the dilution factor, and then multiplying by the total of BALF volume collected.

### Multiplex analysis of inflammatory cytokine levels

We quantified 31 different mediators in the BALF and plasma by using a Discovery Assay® (Mouse Cytokine and Chemokine Array 31-Plex (MD31), Eve Technologies Corp, Calgary, AB, Canada). The multiplex assay was performed at Eve Technologies using the Bio-Plex™ 200 system (Bio-Rad Laboratories, Inc., Hercules, CA, USA) and a Milliplex Mouse Cytokine/Chemokine kit (Millipore, St. Charles, MO, USA) according to Eve Tech protocol (Eve Technologies 2021). The 31-plex consisted of eotaxin, G-CSF, GM-CSF, IFN-γ, IL-1α, IL-1β, IL-2, IL-3, IL-4, IL-5, IL-6, IL-7, IL-9, IL-10, IL-12 (p40), IL-12 (p70), IL-13, IL-15, IL-17, IP-10, KC, LIF (leukemia inhibitory factor), LIX [lipopolysaccharide- induced CXC chemokines (CXCL5)], MCP-1, M-CSF (macrophage colony-stimulating factor), MIG (monokine induced by gamma interferon), MIP-1α, MIP-1β [macrophage inflammatory protein-1β (CCL4)], MIP-2, RANTES [regulated upon activation, normal T cell expressed and presumably secreted (CCL5)], TNF-α (tumor necrosis factor-alpha), and VEGF. The assay sensitivities of these markers range from 0.1 pg/mL to 33.3 pg/mL. Individual analytes’ values and other assay details are available on Eve Technologies’ website and in the Milliplex protocol.

### Histopathological evaluation and lung injury scores

The pulmonary airway was flushed with 0.9% NaCl, and the left lobe was inflated using a 10 mL syringe filled with a fixative (10% NBF) with an attached blunt tip needle (23 gauge). The lung was gently inflated at a 20 cm H_2_O pressure with fixative (10% NBF) until the lobe was fully, uniformly, and consistently expanded (not allowing fixative to ooze through the lung surface). This provided optimal airway expansion without causing tissue disruption. The left lobe was kept in fixative for 48 h, and the 10% NBF was replaced by PBS and stored at 4 °C. The left lung was embedded into paraffin blocks, which were sliced into two longitudinal slices of 5 μm thickness, and each slice were spaced by 50 μm in the middle part of the lung. After embedding and mounting of the tissues, the two slices were stained with Haematoxylin and Eosin (H&E). A blinded histologist evaluated the general morphology of alveolar septa, lung structure, and inflammation according to the general scoring described and adopted by Matute-Bello et al. (2008) (Aeffner et al. 2015).

### Statistical analysis

Results are expressed as means ± SEM. Comparisons were made on normally distributed data using ANOVA (Analysis of Variance), followed by a Fisher *post hoc* test to assess the difference between LPS + vehicle group with GraphPad Prism Software version 8.0 (San Diego, CA, USA). Differences were considered statistically significant when *P* values were less than 0.05.

## Results

**Figure 1.**
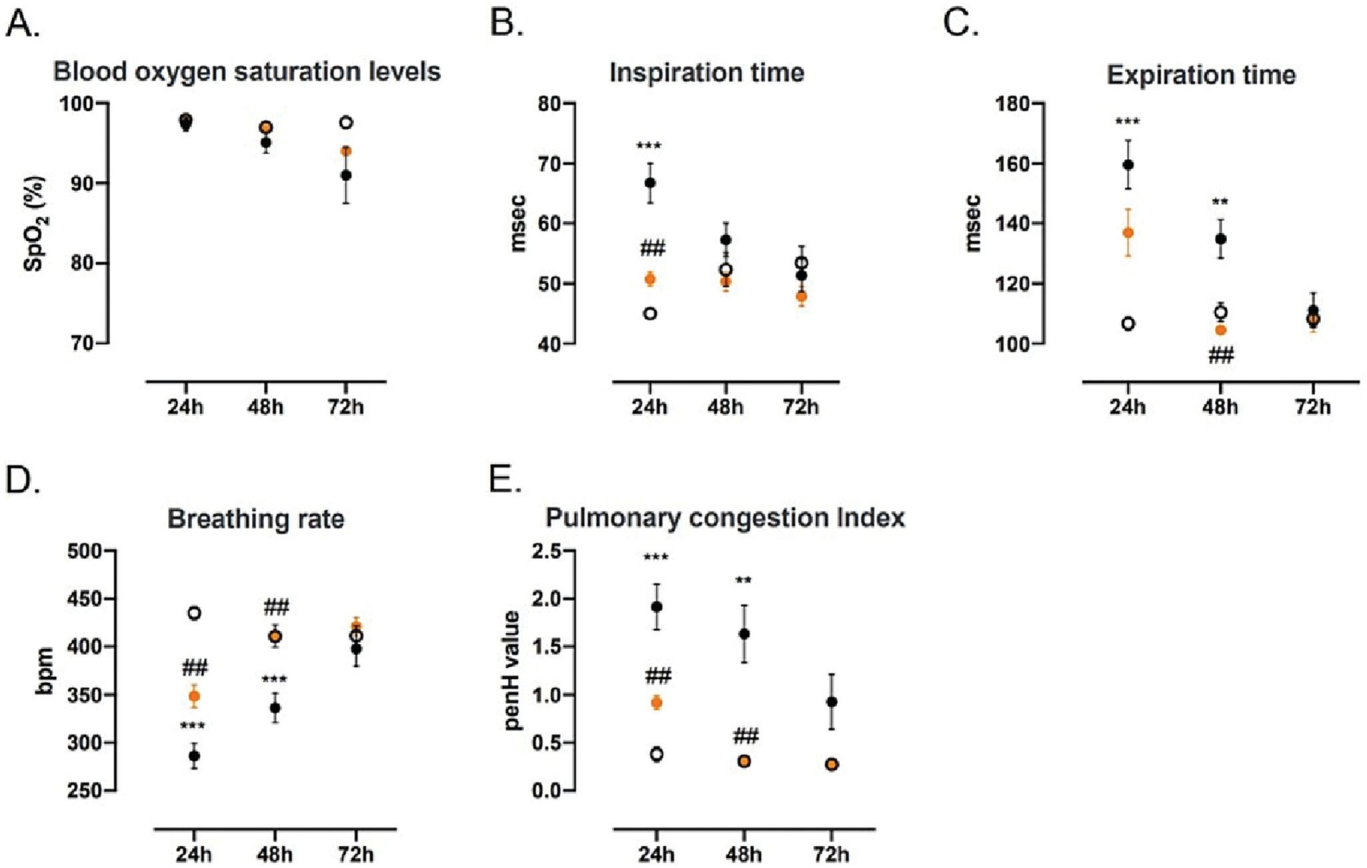
(A) Blood oxygen levels; (B): Inspiration times; (C) Expiration times; (D) Breathing rates; (E) Pulmonary Congestion Indexes at 24, 48 and 72 h, where red dot = mice treated with SPPCT-800 after LPS; black dot = mice treated with LPS and vehicle; open dot = mice receiving sham injections. * Designates differences between sham animals and SPPCT-800 treated animals, and # designates differences between animals in the LPS + vehicle group and SPPCT-800 treated animals (*Signifies *p* < 0.05, **p < 0.01 or *** *p* < 0.001 while # signifies *p* < 0.05, ## *p* < 0.01 or ### *p* < 0.001).

Evidence of SPPCT-800 efficacy against ARDS was found in all three studies. The best results were found in the 72 h study. This is likely due to the fact that mice in the 72 h study received a cumulative dose over the three days of 1600 mg/kg, which is eight times the single ‘high dose’ (200 mg/kg) used in the 24 h studies, although it could also be that the positive effects of SPPCT-800 are not fully seen at 24 h. Figure 1 shows blood oxygen saturation levels, inspiration time, expiration time, breathing rate and pulmonary congestion index (at 24, 48, and 72 h), where red dot = mice treated with SPPCT-800 after LPS; black dot = mice treated with LPS and vehicle; open dot = mice receiving sham injections. * Designates differences between sham animals and SPPCT-800 treated animals, and # designates differences between animals in the LPS + vehicle group and SPPCT-800 treated animals (*Signifies *p* < 0.05, **p < 0.01 or ***p < 0.001 while # signifies *p* < 0.05, ## *p* < 0.01 or ### *p* < 0.001).

### Oxygen saturations

An important measure in ARDS patients is the maintenance of sufficient levels of oxygen in the arterial blood. In our study, SpO_2_ levels were maintained in the SPPCT-800 group compared to the untreated LPS group, where SpO_2_ levels fell. Figure 1(A) shows the %SpO_2_ levels in sham-treated, control and SPPCT-800 treated mice. SpO_2_ levels remained at 98% in the sham-treated mice over the 72 h period of the study. No major changes were seen at 24 h, but at 48 h, SpO_2_ fell to 94% in controls, while remaining at 97% for treated animals. At 72 h, SpO_2_ was 90% for controls, but was maintained at 94% in treated mice.

### Respiratory functions

Improvements after SPPCT-800 treatment were also seen on tests of respiratory function at all measurement times. At 24 h, inspiration times fell from 67 msec in controls to 51 msec in treated animals (*p* < 0.01). The penH value also fell from 1.8 to 0.9 (*p* < 0.01). At 24 h, the expiration time was also decreased, from 160 msec in controls compared to 139 msec in treated mice. At 48 h, expiration time was 137 msec for controls but only 107 msec for treated animals (*p* < 0.01) (Figure 1(B–E)).

### Cell counts and protein levels in BALF

**Figure 2.**
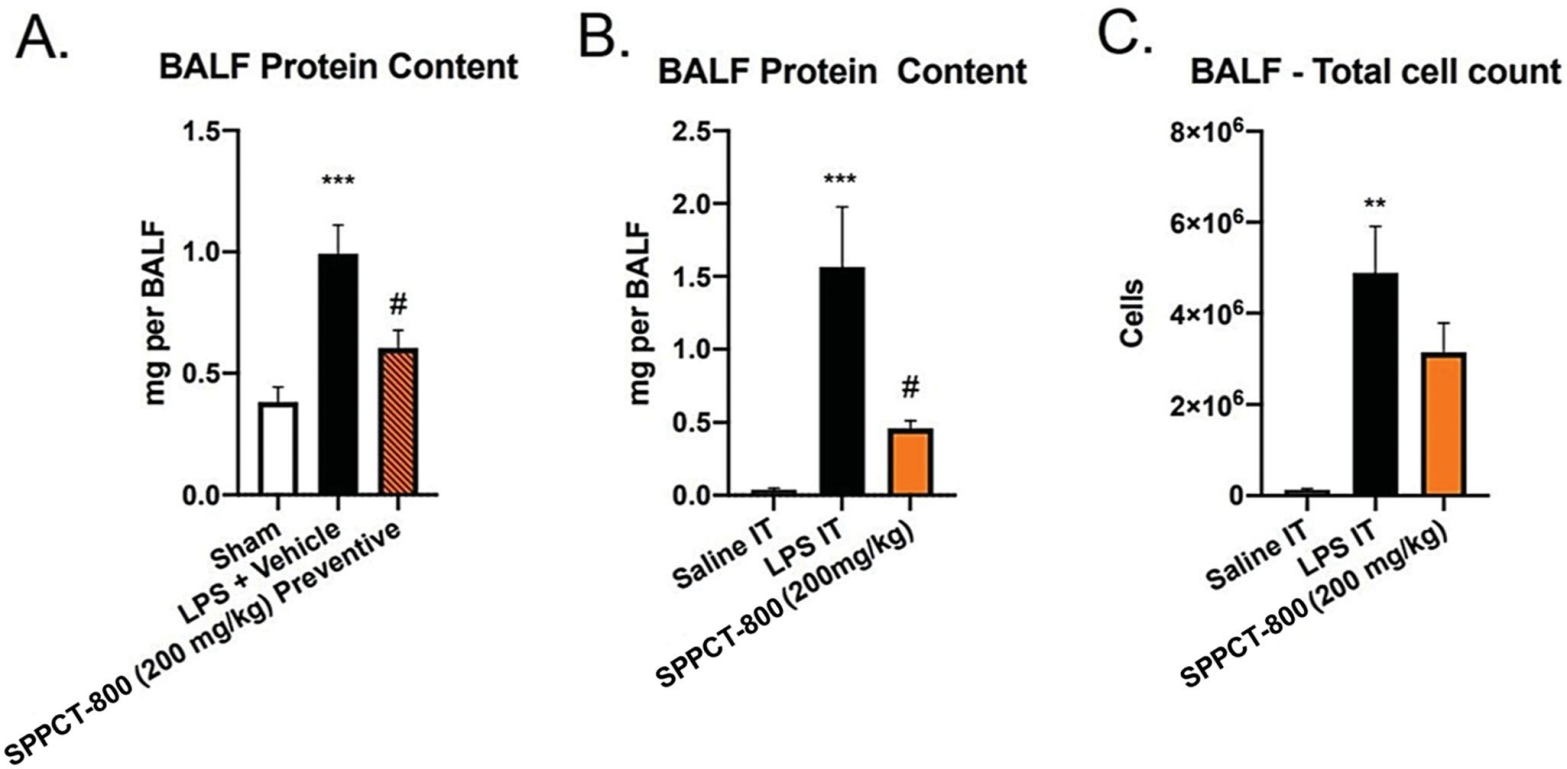
(A) Protein content in BALF at 24 h with SPPCT-800 given one-half h before LPS; (B) with SPPCT-800 given three h after LPS; (C) Total cell count in BALF at 24 h (*Designates differences between sham animals and SPPCT-800 treated animals, and # designates differences between animals in the LPS + vehicle group and SPPCT-800 treated animals. ** means *p* < 0.01 or *** *p* < 0.001 while # signifies *p* < 0.05).

A single dose of SPPCT-800 gave evidence that it could reduce inflammation as early as 24 h after LPS injection. In the therapeutic study, where SPPCT-800 was given 3 h after LPS, BALF protein at 24 h was 1.5 mg in controls, but only 0.4 mg in treated mice (*p <* 0.05). In the preventive study, where SPPCT-800 was given 30 min before LPS, results were similar. BALF protein was 1.0 mg in controls, but only 0.6 mg in treated mice (*p <* 0.05). There were also major reductions in BALF total cell count from 4.7 × 10^6^ cells in control mice compared to 2.9 × 10^6^ cells in treated mice (Figure 2).

### Pro-inflammatory cytokines

Dramatic reductions in the pro-inflammatory cytokines tested were seen after treatment with SPPCT-800, at 24 h and at 72 h, in both the plasma and the BALF (Tables 1 and 2). Major differences between controls and treated mice were seen in levels of TNF-α, INF-γ, G-CSF, GM-CSF, ILs, VEGF, MCP-1, KC and MIP-1α in all three studies. For example, levels of the important cytokine, TNF-α, were greater at 24 h in the plasma in controls, at 36 ± 2 pg/mL than in treated mice, at 14 ± 1 pg/mL (*p* < 0.001), and in the BALF, at 185 ± 31 pg/mL compared to 153 ± 32 pg/mL (*p* = 0.0959). Similarly, in the 72 h study, TNF-α levels were reduced in the plasma from 28 ± 7 pg/mL to 7 ± 2 pg/mL (*p* < 0.05) and in the BALF from 173 ± 51 pg/mL to 62 ± 17 pg/mL (*p* = 0.0613).

**Table 1.** Cytokine levels in plasma measured at 24 h after sham injection, after LPS-vehicle injection, after LPS preceded by SPPCT-800 by 30 min, and after LPS followed 3 h later by SPPCT-800.

		24 h			72 h	
	Sham	LPS-vehicle	SPPCT-800 Pre	SPPCT-800 Thera	LPS-vehicle	SPPCT-800 Thera
Eotaxin	456 ± 125	428 ± 28	719 ± 138	855 ± 228	341 ± 38	464 ± 252
G-CSF	198 ± 29	25015 ± 889	21035 ± 2241	22915 ± 410	5720 ± 2510	1116 ± 502
GM-CSF	6 ± 1	46 ± 3	12 ± 2	11 ± 1	49 ± 9	6 ± 2
IFN γ	1 ± 1	9 ± 2	1 ± 1	1 ± 1	29 ± 13	6 ± 2
IL1-α	101 ± 33	303 ± 26	3196 ± 3107	159 ± 39	161 ± 31	63 ± 27
IL1-β	1.5 ± 0.1	12.7 ± 6.2	3.8 ± 0.7	3.6 ± 0.6	5.3 ± 1.5	6.8 ± 3.8
IL2	6 ± 3	36 ± 7	11 ± 3	16 ± 1	52 ± 11	34 ± 10
IL3	0.4 ± 0.1	2.8 ± 0.8	0.9 ± 0.1	0.5 ± 0.7	1.3 ± 0.6	0.1 ± 0.1
IL4	0.3 ± 0.2	1.5 ± 0.7	0.2 ± 0.1	0.4 ± 0.2	5.9 ± 4.4	0.6 ± 0.4
IL5	4 ± 1	32 ± 5	8 ± 2	11 ± 2	22 ± 5	2 ± 1
IL6	2 ± 1	641 ± 228	176 ± 29	155 ± 30	539 ± 213	76 ± 31
IL7	3 ± 1	34 ± 25	8 ± 2	4 ± 1	7 ± 1	3 ± 1
IL9	8 ± 2	52 ± 8	2 ± 1	6 ± 2	46 ± 18	3 ± 1
IL10	4 ± 1	128 ± 23	57 ± 8	90 ± 32	40 ± 17	5 ± 1
IL12 (p40)	7 ± 2	21 ± 3	12 ± 1	12 ± 2	12 ± 1	8 ± 1
IL12 (p70)	8 ± 2	37 ± 15	17 ± 7	40 ± 19	98 ± 71	11 ± 5
IL13	16 ± 1	72 ± 6	22 ± 3	22 ± 2	72 ± 16	23 ± 3
IL15	19 ± 14	408 ± 243	63 ± 15	49 ± 16	124 ± 17	40 ± 22
IL17	1.0 ± 0.2	14.2 ± 10.4	2.6 ± 0.3	2.3 ± 0.6	11 ± 4	4 ± 3
IP-10	63 ± 6	504 ± 52	790 ± 206	605 ± 102	300 ± 66	150 ± 34
KC	51 ± 21	3933 ± 1362	1271 ± 224	764 ± 274	1049 ± 358	502 ± 150
LIF	0.6 ± 0.1	10.5 ± 8.2	1.3 ± 0.2	1.5 ± 0.2	2.2 ± 0.7	0.5 ± 0.1
LIX	71 ± 23	3067 ± 874	470 ± 145	547 ± 203	2117 ± 529	574 ± 445
MCP-1	11 ± 1	157 ± 22	86 ± 20	157 ± 62	55 ± 7	34 ± 6
M-CSF	6 ± 1	42 ± 4	12 ± 1	16 ± 4	24 ± 1	5 ± 2
MIG	557 ± 201	1261 ± 121	2486 ± 203	2593 ± 317	353 ± 122	447 ± 137
MIP-1α	38 ± 5	197 ± 28	88 ± 4	92 ± 7	210 ± 14	133 ± 11
MIP-1β	44 ± 7	121 ± 11	109 ± 8	120 ± 29	102 ± 16	55 ± 13
MIP-2	91 ± 7	86 ± 13	138 ± 7	128 ± 5	61 ± 8	56 ± 17
RANTES	15 ± 4	360 ± 45	200 ± 28	270 ± 70	64 ± 13	19 ± 2
TNF-α	5 ± 1	36 ± 2	14 ± 1	14 ± 1	28 ± 7	7 ± 2
VEGF	0.4 ± 0.1	1.3 ± 0.3	0.5 ± 0.1	0.6 ± 0.1	28.0 ± 7.0	7.4 ± 2.4

Cytokine levels in plasma measured at 72 h after LPS + vehicle injection, and after LPS injection followed by eight SPPCT-800 injections over 72 h.

**Table 2.** Cytokine levels in BALF measured at 24 h after sham injection, after LPS-vehicle injection, after LPS preceded by SPPCT-800 by 30 min, and after LPS followed 3 h later by SPPCT-800.

		24 h			72 h	
	Sham	LPS-vehicle	SPPCT-800 Pre	SPPCT-800 Thera	LPS-vehicle	SPPCT-800 Thera
Eotaxin	3 ± 1	15 ± 3	10 ± 1	14 ± 3	24 ± 8	6 ± 2
G-CSF	39 ± 8	8034 ± 699	6468 ± 381	7060 ± 853	3648 ± 1039	1233 ± 572
GM-CSF	16 ± 1	81 ± 6	26 ± 5	46 ± 9	48 ± 5	9 ± 1
IFN γ	7 ± 1	10 ± 1	2 ± 1	3 ± 1	34 ± 14	12 ± 1
IL1-α	38 ± 3	220 ± 12	183 ± 25	331 ± 67	494 ± 153	576 ± 169
IL1-β	1 ± 1	190 ± 82	69 ± 9	103 ± 18	174 ± 42	89 ± 24
IL2	5.5 ± 0.3	9.6 ± 0.5	1.5 ± 0.2	1.5 ± 0.3	9.2 ± 2.1	1.7 ± 0.4
IL3	1.4 ± 0.1	3.2 ± 0.3	0.4 ± 0.1	0.5 ± 0.1	3.5 ± 0.8	0.5 ± 0.2
IL4	0.06 ± 0.03	0.36 ± 0.06	0.08 ± 0.01	0.10 ± 0.01	0.74 ± 0.16	0.24 ± 0.05
IL5	29 ± 6	39 ± 6	10 ± 3	10 ± 2	12 ± 4	0.4 ± 0.1
IL6	11 ± 3	942 ± 194	856 ± 188	850 ± 212	4664 ± 2591	276 ± 151
IL7	2.8 ± 0.2	3.7 ± 0.2	1.7 ± 0.3	1.7 ± 0.2	3.6 ± 0.6	2.0 ± 0.5
IL9	9.8 ± 1.1	14.5 ± 1.3	7.0 ± 0.3	8.8 ± 1.3	16.7 ± 3.3	8.5 ± 1.6
IL10	1.2 ± 0.1	3.5 ± 0.4	1.4 ± 0.3	1.4 ± 0.3	9.0 ± 6.7	1.1 ± 0.5
IL12 (p40)	1.2 ± 0.2	3.8 ± 0.3	1.6 ± 0.3	2.0 ± 0.3	6.2 ± 2.0	6.0 ± 1.2
IL12 (p70)	9.5 ± 0.9	15.6 ± 1.8	7.4 ± 1.0	6.0 ± 1.0	26.5 ± 6.8	15.7 ± 3.9
IL13	0.5 ± 0.1	4.4 ± 0.5	0.7 ± 0.1	1.1 ± 0.2	7.4 ± 2.1	1.1 ± 0.2
IL15	9.7 ± 1.4	16.8 ± 1.5	5.1 ± 0.8	3.1 ± 0.7	12.8 ± 3.8	4.8 ± 2.6
IL17	0.2 ± 0.1	10.6 ± 2.7	8.3 ± 3.2	13.6 ± 5.6	102.9 ± 75.6	53.2 ± 26.9
IP-10	9 ± 1	115 ± 13	125 ± 10	163 ± 33	1061 ± 339	1583 ± 385
KC	20 ± 2	932 ± 105	622 ± 89	1017 ± 140	714 ± 143	216 ± 15
LIF	2 ± 1	39 ± 8	38 ± 8	39 ± 12	103 ± 55	11 ± 7
LIX	3 ± 1	74 ± 5	94 ± 6	67 ± 20	91 ± 24	25 ± 18
MCP-1	10 ± 1	372 ± 75	462 ± 103	494 ± 103	624 ± 461	84 ± 34
M-CSF	2 ± 1	30 ± 5	21 ± 3	27 ± 6	21 ± 5	11 ± 1
MIG	3.4 ± 1.0	16.7 ± 4.0	6.8 ± 1.5	6.6 ± 2.0	346.8 ± 212.7	749.7 ± 186.7
MIP-1α	18 ± 3	447 ± 58	323 ± 52	564 ± 132	1082 ± 274	1676 ± 554
MIP-1β	19 ± 1	536 ± 105	801 ± 208	1924 ± 812	1138 ± 513	1561 ± 440
MIP-2	33 ± 3	1410 ± 950	771 ± 107	1054 ± 106	3647 ± 2324	2649 ± 898
RANTES	2 ± 1	30 ± 3	45 ± 6	46 ± 6	38.17 ± 12	57 ± 20
TNF-α	1 ± 1	185 ± 31	91 ± 32	153 ± 32	173 ± 51	62 ± 17
VEGF	13 ± 2	41 ± 3	33 ± 2	34 ± 2	29 ± 7	11 ± 3

Cytokine levels in BALF measured at 72 h after LPS + vehicle injection, and after LPS injection followed by eight SPPCT-800 injections over 72 h.

SPPCT-800 profoundly depressed levels of another key cytokine, IFN-γ, at 24 h in the plasma (*p* < 0.01), and in the BALF (*p* < 0.001). Large differences in IFN-γ levels were also seen at 72 h, in both the plasma and the BALF. GM-CSF, which causes the secretion of neutrophils, monocytes and other cells, and is, thus, thought to play a major role in causing lung damage in ARDS patients, was markedly reduced in all mice treated with SPPCT-800. At 24 h, there were highly significant differences in GM-CSF levels between controls and treated groups in both the plasma (46 ± 3 to 11.1 ± 1 pg/mL) and the BALF (81 ± 6 to 46 ± 9 pg/mL) (*p* < 0.001). Likewise, at 72 h, significant differences in GM-CSF levels were noted in both the plasma (49 ± 9 to 6 ± 2 pg/mL) (*p* < 0.01) and the BALF (48 ± 5 to 9 ± 1 pg/mL) (*p* < 0.001).

Of the 15 interleukins tested, major reductions with SPPCT-800 were consistently noted in 14 of the treated animals. At 24 h, IL-1β, IL-2 (*p* < 0.05), IL-3 (*p* = 0.0859 in 200 mg therapeutic group), IL-4, IL-5 (*p* < 0.05), IL-6 (*p* = 0.0624), IL-7, IL-9 (*p* < 0.001), IL-10 (*p* = 0.0767 in the preventive group), IL-12 (p 40) (*p* = 0.1101 in the preventive group), IL-12 (p70), IL-13 (*p* < 0.001), IL-15 and IL-17 were markedly reduced. SPPCT-800 did not reduce levels of plasma IL-1α in any of the mouse groups at 24 h. Similar major reductions in these interleukins at 24 h were seen in the BALF, with major reductions in IL-1β, IL-2 (*p* < 0.001), IL-3 (*p* < 0.001), IL-4 (*p* < 0.05), IL-5 (*p* < 0.01), IL-7 (*p* < 0.001), IL-9 (*p* < 0.01), IL-10 (*p* < 0.01), IL-12 (p40) (*p* < 0.001), IL-12 (p70) (*p* < 0.01), IL-13 (*p* < 0.001) and IL-15 (*p* < 0.001). SPPCT-800 also suppressed G-CSF, VEGF, KC, LIF (leukemia inhibitory factor), LIX (LPS-induced CXC chemokine 5), and M-CSF (macrophage colony stimulating factor), in the plasma and the BALF. MCP-1 was inhibited by SPPCT-800 in the plasma at 24 h, and in both the plasma and BALF at 72 h. Suppressive effects against MIP-1α and MIP 1-β (macrophage inflammatory protein 1-β) were found at 24 h in both the plasma and BALF and also at 72 h in the plasma. RANTES (regulated on activation, normal t cell expressed and secreted; CCL5) was suppressed in the plasma at both 24 h and 72 h, but not in the BALF. Results were mixed, with marked suppression by SPPCT-800 in the plasma or the BALF, but not in both, against MIG, IP10 and eotaxin, an eosinophil chemotactic protein (Tables 1 and 2).

### Body weight loss

**Figure 3.**
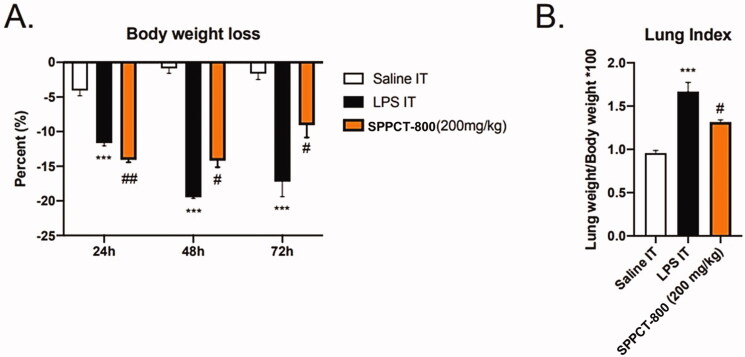
(A) Body weight loss is significantly reduced at 48 h and 72 h after LPS treatment in animals treated with SPPCT-800; (B) The lung weight/body weight ratio is reduced (*Designates differences between sham animals and SPPCT-800 treated animals, and # designates differences between animals in the LPS + vehicle group and SPPCT-800 treated animals. *** signifies *p* < 0.001 while # signifies *p* < 0.05 and ## *p* < 0.01).

An important measure of a treatment’s efficacy is the degree it reduces the weight loss in the animals with ARDS. At 24 h, both the LPS mice and the LPS mice treated with SPPCT-800 showed a major loss of body weight compared to sham mice, with no protective effect from SPPCT-800 at that time (14% weight loss with SPPCT-800 compared to 12% with LPS alone). The weight loss at 48 h stabilized at 14% in the SPPCT-800 group, while it reached 20% in the untreated LPS group (*p* < 0.05). Importantly, at 72 h, body weight loss decreased by 8% with SPPCT-800, compared to a loss of 18% in the untreated LPS group (*p* < 0.05). SPPCT-800 also tended to reduce the lung weight for animals sacrificed at 72 h (*p* = 0.1100). Thus, it significantly decreased the lung index (lung weight/body weight × 100; *p* < 0.05), and decreased the wet lung weight/dry lung ratio (Figure 3).

### Histological evaluation and lung injury scores

**Figure 4.**
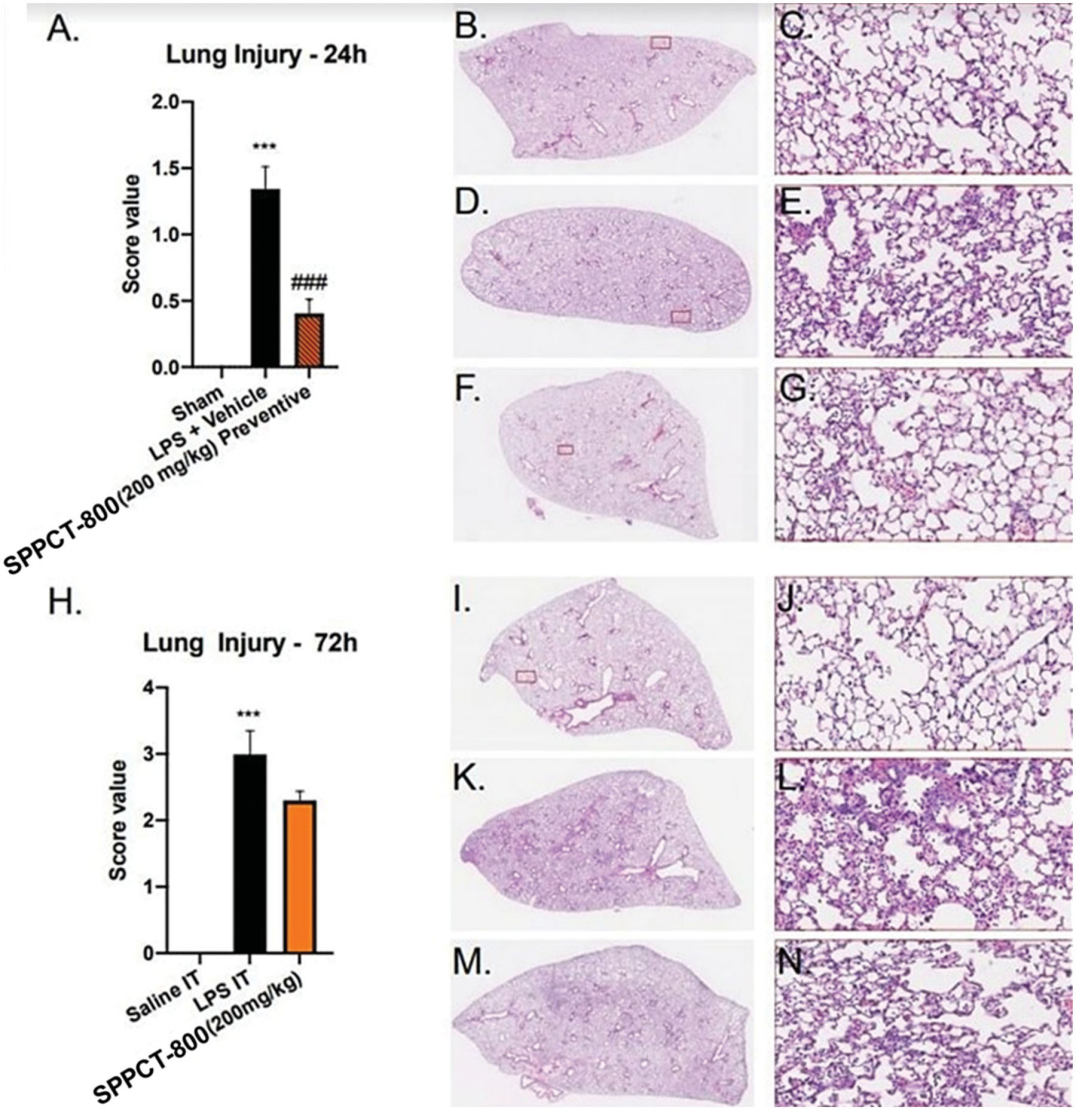
(A) Lung injury scores at 24 h; (B + C) Histology at 24 h in sham mice; (D + E) Histology at 24 h in mice that received LPS + vehicle; (F + G) Histology at 24 h in mice that received SPPCT-800 as a preventative; H) Lung injury scores at 72 h; (I + J) Histology at 72 h in sham mice; (K + L) Histology at 72 h in mice that received LPS + vehicle; (M + N) Histology at 72 h in mice that received SPPCT-800 (*Designates differences between sham animals and SPPCT-800 treated animals, and # designates differences between animals in the LPS + vehicle group and SPPCT-800 treated animals. *** signifies *p* < 0.001 while ### signifies *p* < 0.001).

Histological analysis, done as early as 24 h, showed SPPCT-800 could reduce the injury to the lung induced by LPS. SPPCT-800, given 30 min prior to LPS as a preventive, significantly reduced the lung injury score value. The lung injury score at 24 h was 1.4 when mice were given LPS alone, but was only 0.3 when SPPCT-800 was given as a preventative (*p* < 0.01). At 72 h, the lung injury score was also reduced. Mice in the multi-dose SPPCT-800 treatment group had a reduced score of 2.2 compared to a score of 3.0 in the mice not given SPPCT-800 (Figure 4).

## Discussion

In this study, the potential protective and therapeutic effects of SPPCT-800 against ARDS were shown by multiple measures. The mice treated with this agent demonstrated higher oxygen saturation levels, and better results on pulmonary function testing. Protein content and neutrophil cell counts in the BALF were reduced. Inflammation was profoundly suppressed in the animals treated with SPPCT-800. The key cytokines, TNF-α, crucial in the aetiology of numerous diseases, and interferon γ, central for its capacity to induce indoleamine 2,3-dioxygenase (Sordillo and Sordillo 2019), as well as almost all interleukins, were profoundly suppressed. Levels of VEGF, thought to play a major role in the massive lung damage and lung edoema seen with ARDS, were markedly reduced (Barratt et al. 2014). G-CSF and GM-CSF, which are used to stimulate neutrophils in patients receiving chemotherapy, but which can induce an ARDS-like syndrome (Takatsuka et al. 2002; Rhee et al. 2009; Kudlak et al. 2013; Inokuchi et al. 2015), were also dramatically suppressed. A major decrease in weight loss was seen in treated animals compared to controls on days 2 and 3 after therapy. That this effect was not seen on day 1 is consistent with data in animal models that signs of ARDS-induced injury usually begin to appear within 48 h, but that there may be a delay of one day or more before signs of treatment efficacy may be seen (Chen et al. 2010; Aeffner et al. 2015).

Increased inflammation is a crucial part of the development of many diseases, including cancer (Greten and Grivennikov 2019; Sordillo and Sordillo 2021a), Parkinson’s disease (Ferrari and Tarelli 2011) and coronary heart disease (Stanciu 2019), much as the cerebral damage after major neurologic injuries may be due to a massive accumulation of cytokines within the brain (Sordillo et al. 2016). ARDS can be thought of similarly, as a massive accumulation of cytokines within the lungs. Sepsis, itself, can be interpreted as a whole-body inflammatory process brought on by systemic infections. Thus, multiple studies have been done in ARDS patients with medications that suppress cytokines. Clinical trials with anti-inflammatory agents have given equivocal results, and many of these medications subject patients to additional toxicities (Dushianthan et al. 2011; Boyle et al. 2013; Koh 2014; Patel et al. 2018). Corticosteroids have been the most commonly used treatment in patients with ARDS, although many new treatments, including antivirals, are being introduced to treat the subset of patients with ARDS secondary to SARS-COVID-19. Again, treatment with corticosteroids is controversial, has not been proven to increase survival, and exposes the patient to numerous additional risks, including severe hyperglycaemia, hypokalaemia, GI bleeding, severe hypertension, and fungal or bacterial infections (Schäcke et al. 2002; Zhang et al. 2015; Villar et al. 2020).

SPPCT-800, with chemical formula [C_42_H_78_O_12_P]_2 _Mg, is a white crystalline powder which can be taken orally. It has detectable activity after a single dose of 10 mg/kg, and is non-toxic at doses of up to 800 mg/kg per day. We believe SPPCT-800 was effective in this study because of its ability to suppress profoundly multiple proinflammatory cytokines. Cytokine suppression is known to be an effective strategy for the treatment of many diseases (Leppkes and Neurath 2020; Sordillo et al. 2016).

SPPCT-800 has not been investigated in human patients, but has been studied extensively in multiple animal species as a potential treatment for cardiac diseases. This study demonstrates that, in the murine LPS-induced ARDS model, a single dose of 200 mg/kg of SPPCT-800, given 30 min before LPS challenge, or 3 h after LPS, could modify toxic effects of ARDS such as weight loss, and can reduce severe damage to the lung by suppressing inflammation. Further studies on the effect of this unique lipid compound against ARDS are indicated, particularly when given for extended periods or with higher cumulative doses.
